# A Novel SLC9A3R1 Mutation as a Rare Cause of Infantile Hypercalcemia

**DOI:** 10.7759/cureus.102923

**Published:** 2026-02-03

**Authors:** Padala Ravi Kumar, Ankeet Biswas, Deepak K Dash, Debasish Patro, D. Sai Madhav Reddy

**Affiliations:** 1 Department of Endocrinology, MKCG Medical College and Hospital, Berhampur, IND

**Keywords:** genetic testing, hypercalcemia, nphlop2, slc9a3r1, zoledronic acid

## Abstract

Hypercalcemia in infants is a rare but potentially serious condition characterized by elevated serum calcium levels. We report a case of a two-month-old female presenting with poor feeding, lethargy, irritability, and failure to thrive, and she was found to have hypercalcemia. On examination, she had a weight of 3.1 kg (-3.94 SDS) with normal facial features. Laboratory investigations revealed elevated serum calcium, low phosphate, suppressed parathyroid hormone *(*PTH), and high 1, 25-dihydroxyvitamin D levels. Renal ultrasonography revealed bilateral medullary nephrocalcinosis. Clinical exome sequencing identified a heterozygous missense variant in the SLC9A3R1 gene, leading to a diagnosis of hypophosphatemic nephrolithiasis/osteoporosis-2 (NPHLOP2). She was initially managed with calcium restricted diet, intravenous fluid rehydration, and administration of zoledronic acid. On follow-up at one month, the patient showed significant symptomatic improvement with normalization of serum calcium levels along with weight gain. This case highlights the role of genetic testing to identify rare genetic causes of hypercalcemia during infancy. Early diagnosis and appropriate management of NPHLOP2 can significantly improve an individual's outcomes and quality of life.

## Introduction

Hypercalcemia in an infant is defined as a serum calcium level that exceeds the upper limit of the normal range for newborns [1]. The upper limit of serum calcium in an infant is 11.3 mg/dL (2.83 mmol/L) [2,3]. It is crucial to understand that the presentation of hypercalcemia in a child can vary widely, from asymptomatic cases to severe clinical manifestations. The signs and symptoms of hypercalcemia include lethargy, poor feeding, failure to thrive, dehydration, hypertension, altered mental status, hypotonia, irritability, seizure, cardiac arrhythmias, nephrocalcinosis, and gastrointestinal symptoms such as constipation, abdominal pain, or vomiting. The causes of hypercalcemia in children can be divided into parathyroid hormone (PTH)-dependent and PTH-independent. PTH-independent hypercalcemia is more common in children than PTH-dependent hypercalcemia [4]. Causes of PTH-dependent hypercalcemia include primary hyperparathyroidism (PHPT), tertiary hyperparathyroidism, familial hypocalciuric hypercalcemia, etc. Acquired etiologies of PTH-dependent hypercalcemia in neonates include maternal hypocalcemia and extracorporeal membrane oxygenation [4]. Congenital syndromes that present with PTH-independent hypercalcemia include idiopathic infantile hypercalcemia (IIH), Williams syndrome, blue diaper syndrome, hypophosphatasia, and other inborn errors of metabolism [1]. The acquired causes of PTH-independent hypercalcemia in children include vitamin D overdose, granulomatous disorders, and endocrine disorders, such as pheochromocytoma, Addison's disease, thyrotoxicosis, and severe congenital hypothyroidism [4]. In infancy, the acquired causes of PTH-independent hypercalcemia are more common than the genetic causes. Although genetically mediated PTH-independent hypercalcemia is relatively uncommon, it should be considered in infants with severe, persistent, or recurrent hypercalcemia, particularly when occurring early in life. Hence, clinical exome sequencing is crucial to diagnose the genetic causes of infantile hypercalcemia.

IIH is most commonly attributed to biallelic mutations in the CYP24A1 or SLC34A1 genes, both of which result in elevated levels of 1,25-dihydroxyvitamin D and hypercalcemia, accompanied by suppression of PTH secretion [4,5]. More recently, attention has turned to rare variants in the SLC9A3R1 gene, which encodes the Na+/H+ exchange regulatory factor 1 (NHERF1). NHERF1 is a cytoplasmic scaffold protein that plays a pivotal role in connecting the plasma membrane proteins with members of the erzin/moeisin/radixin family, helping to link them to the actin cytoskeleton, facilitating their surface expression. NHERF1 also promotes renal phosphate reabsorption through its interaction with sodium-phosphate co-transporters in the proximal tubular cells of the kidney [6]. Loss of function of NHERF1 leads to renal phosphate wasting, which further leads to upregulation of 1α hydroxylase and elevated levels of 1,25-dihydroxyvitamin D. The elevated 1,25-dihydroxyvitamin D is responsible for hypercalcemia and subsequent hypercalciuria, leading to nephrocalcinosis [4].

SLC9A3R1 mutations are a rare but important cause of infantile hypercalcemia, which has been reported in 64 cases to date, with 17 germline mutations as per the ClinVar database of NCBI [7]. Here, we report a novel mutation in the SLC9A3R1 gene in a two-month-old infant.

## Case presentation

A two-month-old female child presented to the Paediatrics Emergency Department with poor feeding, lethargy, irritability, and failure to thrive for one month. She was born at term by normal vaginal delivery after an uncomplicated pregnancy, with a birth weight of 3.1 kg. The infant was exclusively breastfed and received vitamin D supplementation at a dose of 400 IU daily as per recommendations of the Indian Academy of Paediatrics (IAP). She also received calcium supplementation (150 mg/day) at the age of one month for a brief period of one week, after which it was stopped. No further calcium supplementation was given till two months of age. At the age of two months, hypercalcemia (mean serum calcium: 3.59 mmol/L) and hypophosphatemia (mean serum phosphate: 1.39 mmol/L) were detected. Multiple serum calcium and phosphate measurements confirmed hypercalcemia and hypophosphatemia. Thereafter, the case was referred to the Department of Endocrinology for further work-up.

Examination: She was found to have a length of 54 cm (-1.55 SDS) and a weight of 3.1 kg (-3.94 SDS). Physical examination revealed lethargy and dehydration, with decreased skin turgor and dry mucous membranes. No dysmorphic facial features were noted. Blood pressure was 88/52 mmHg in the right arm supine position (50th centile for age and sex). Cardiovascular examination was normal without any murmur on auscultation.

Laboratory work-up: Laboratory investigations revealed a raised serum calcium level of 3.59 mmol/L (normal range: 2.17-2.82 mmol/L), low serum phosphate level of 1.39 mmol/L (normal range for age: 1.68-2.71 mmol/L), serum albumin of 35 g/L, blood urea level of 6.66 mmol/L, and serum creatinine level of 35 µmol/L (Table 1). Arterial blood gas analysis revealed normal pH, ruling out renal tubular acidosis. The intact parathyroid hormone (iPTH) level was suppressed with a value of 0.77 pmol/L (normal range: 1.7-6.9 pmol/L). The 25-hydroxyvitamin D level was normal with a value of 148.92 nmol/L (normal range: 75-250 nmol/L), and the 1,25-dihydroxyvitamin D level was significantly elevated with a value of 403 pmol/L (normal range: 47.76-190.32 pmol/L). Serum sodium, potassium, glucose, magnesium, complete blood count, and serum TSH were within normal limits. Spot urine calcium to creatinine ratio was 2.32, suggesting hypercalciuria. TMP-GFR was low with a value of 1.21 mmol/L (normal range: 1.43-3.43). Evaluation for PTH-independent causes of hypercalcemia was done. ESR was 08 mm fall in the first hour, and the Mantoux test was negative, ruling out tuberculosis. The serum ACE level was 25 U/L (normal: 8-52 U/L), making sarcoidosis unlikely in this case. Hypercalcemia associated with malignancy is less likely as the serum PTHrP level was normal (112 pmol/L).

**Table 1:** Results of biochemical, hematological, and hormonal testing Abbreviations: iPTH, intact parathyroid hormone; TSH, thyroid-stimulating hormone; ESR, erythrocyte sedimentation rate; ACE, angiotensin-converting enzyme; PTHrP, parathyroid hormone-related protein

Test	Result	Reference range
Serum calcium	3.59 mmol/L	2.17–2.82 mmol/L
Serum phosphate	1.39 mmol/L	1.68–2.71 mmol/L
Serum albumin	35 g/L	35–55 g/L
ALP	578 U/L	355-1037 U/L
Serum urea	6.66 mmol/L	2.5–6.7 mmol/L
Serum creatinine	35 µmol/L	18–35 µmol/L
iPTH	0.77 pmol/L	1.7–6.9 pmol/L
25-hydroxyvitamin D	148.9 nmol/L	75–250 nmol/L
1,25-dihydroxyvitamin D	403 pmol/L	47.8–190.3 pmol/L
Serum sodium	142 mmol/L	135–145 mmol/L
Serum potassium	4.2 mmol/L	3.5–5.5 mmol/L
Random plasma glucose	4.6 mmol/L	3.6–5.5 mmol/L
Serum magnesium	0.66 mmol/L	0.66–1.07 mmol/L
Hemoglobin	122 g/L	90–140 g/L
Serum TSH	2.70 mIU/L	0.35–5.5 mIU/L
ESR (1 hour)	8 mm/h	<10 mm/h
Serum ACE	25 U/L	8–52 U/L
Serum PTHrP	112 pmol/L	53–298 pmol/L

**Figure 1:**
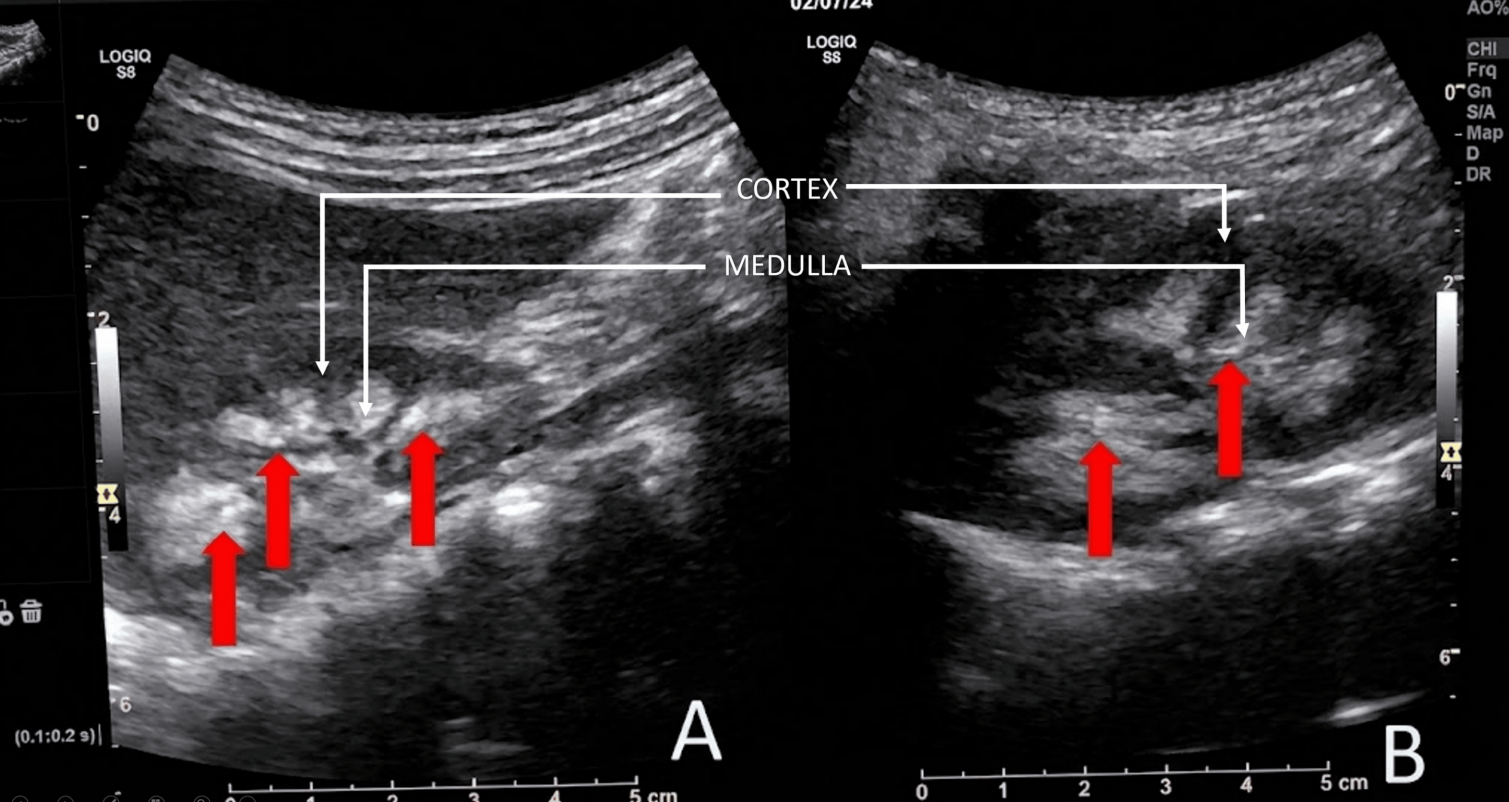
B-mode ultrasonography of the kidneys B-mode ultrasonography of the kidneys using a 4 MHz probe showing (A) the right kidney and (B) the left kidney, with red arrows indicating the areas of medullary nephrocalcinosis.

Imaging: Renal ultrasonography using a 4 MHz probe revealed bilateral medullary nephrocalcinosis (Figure 1), and 2-D echocardiography was normal.

Hence, we suspected idiopathic infantile hypercalcemia, and a blood sample for a genetic test was sent.

Genetic testing: Genetic testing with clinical exome sequencing identified a heterozygous missense variant in exon 4 of the SLC9A3R1 gene (chr17:g.74766944C>T; depth: 185x), classified as a variant of unknown significance and associated with autosomal dominant hypophosphatemic nephrolithiasis/osteoporosis-2 (Table 2). The in silico analysis of this variant predicted it to be damaging on PolyPhen-2 (HumDiv) but requires clinical correlation.

**Table 2:** Genetic analysis report Clinical exome sequencing showing a heterozygous missense variant in exon 4 of SLC9A3R1 (chromosome 17: c.766C>T; genomic position chr17:g.74766944C>T; sequencing depth 185×), resulting in the substitution of serine for proline at codon 256 (p.Pro256Ser; transcript ENST00000262613.10) and associated with autosomal dominant hypophosphatemic nephrolithiasis/osteoporosis type 2.

Gene (Transcript)	Location	Variant	Zygosity	Disease (OMIM)	Inheritance	Classification
SLC9A3R1 (+) (ENST00000262613.10)	Exon 4	c.766C>T (p.Pro256Ser)	Heterozygous	Hypophosphatemic nephrolithiasis/osteoporosis–2 (OMIM #612287)	Autosomal dominant	Uncertain significance (PM2, PP3)

With the above clinical, biochemical, radiological, and genetic evaluation, a diagnosis of hypophosphatemic nephrolithiasis/steoporosis-2 (NPHLOP2) (OMIM #612287) was made.

Treatment: Vitamin D supplementation was discontinued. The patient was rehydrated with 0.9% normal saline (200 mL/m^2^/hr) for three days, while breastfeeding was continued. Despite these measures, her serum calcium levels remained elevated (Table 3). She was subsequently treated with intravenous zoledronic acid at a dose of 0.08 mg (0.025 mg/kg) infused over one hour. Two days after zoledronic acid administration, the patient developed transient hypocalcemia, which was mild, asymptomatic, and self-limiting without the requirement of calcium supplementation. It resolved spontaneously within the next two days. At the time of discharge after 14 days, her serum calcium level was 2.45 mmol/L. During her hospital stay of two weeks, she gained 480 g of weight.

**Table 3:** Trends of serum calcium and phosphate during hospital stay This table depicts the trends of serum calcium and phosphate during hospital stay with initial high levels of calcium and low levels of phosphate. Subsequent to the administration of zoledronic acid on day seven, there was drastic fall in serum calcium level (transient hypocalcemia) and further fall in serum phosphate level, both of which resolved spontaneously within next two days.

Day	Serum Calcium (mmol/L)	Serum Phosphate (mmol/L)
Day 1	3.19	1.26
Day 3	3.44	1.39
Day 5	3.74	1.52
Day 7	3.54	1.61
Day 9	1.97	1.07
Day 11	2.54	1.39
Day 14	2.45	1.36

Follow-up: At one-month follow-up, the patient was active and had gained 1.1 kg (weight: 4.2 kg (-2.86 SDS)). Her serum calcium level was normal (2.39 mmol/L), but serum phosphate was still low (0.9 mmol/L). Repeat ultrasonography of the abdomen and pelvis showed no progression of nephrolithiasis.

Phosphate supplementation was started at a dose of 2 mmol/kg/day in two divided doses. On follow-up at one year and four months, she further gained length of 27 cm and weight of 6.9 kg. Her repeat serum calcium was 2.42 mmol/L, and serum phosphate was 1.54 mmol/L, both of which were normal. Phosphate supplementation was continued at 2 mmol/kg/day in two divided doses.

## Discussion

The present study reports a case of PTH-independent hypercalcemia in a two-month-old child with hypophosphatemia and nephrocalcinosis due to a novel heterozygous mutation in the SLC9A3R1 gene on chromosome 17. She was managed successfully with intravenous zoledronic acid therapy and oral phosphate supplementation.

Infantile hypercalcemia is caused mainly by PTH-independent causes from acquired etiologies, and genetic causes are rare. Idiopathic infantile hypercalcemia (IHH) is the most common genetic cause of infantile hypercalcemia, mostly attributed to biallelic mutations in CYP24A1 or SLC34A1 genes [4]. Mutations in the SLC9A3R1 gene leading to NPHLOP2 are a rare but important cause of infantile hypercalcemia. Table 4 lists a few of these mutations [6,8-10]. NPHLOP2 is characterized by renal phosphate wasting, hypophosphatemia, hypercalcemia, hypercalciuria, and recurrent nephrolithiasis.

**Table 4:** SLC9A3R1 (NHERF1) variants associated with NPHLOP2 These are a few of the identified mutations in SLC9A3R1 responsible for the development of NPHLOP2. Abbreviation: NPHLOP2, nephrolithiasis/osteoporosis-2

Variant (DNA/protein)	Exon	dbSNP number	ClinVar Classification	Condition	Publications	PolyPhen-2
c.328C>G; p.Leu110Val (L110V) [6, 8]	Exon 1	rs35910969	Conflicting: Benign (or) Uncertain	NPHLOP2	Reported in 2 patients with phosphate wasting; nephrolithiasis and low BMD	Benign with a score of 0.073 (sensitivity: 0.93; specificity: 0.84)
c.458G>A; p.Arg153Gln (R153Q) [6, 8]	Exon 2	rs41282065	Conflicting: Likely benign (or) Uncertain	NPHLOP2	Segregates with 3 family members with low phosphate and nephrolithiasis	Probably damaging with a score of 1.000 (sensitivity: 0.00; specificity: 1.00)
c.616T>C; p.Cys206Arg (C206R) [6]	Exon 3	Not available	Not reported	NPHLOP2	Not reported	Benign with a score of 0.098 (sensitivity: 0.93; specificity: 0.85)
c.722A>G; p.Lys241Arg (K241R) [6]	Exon 3	rs373061379	Not reported	NPHLOP2	Not reported	Benign with a score of 0.001 (sensitivity: 0.99; specificity: 0.15)
c.673G>A; p.Glu225Lys (E225K) [8, 9, 10]	Exon 3	rs119486097	Benign (or) Likely benign	NPHLOP2	Reported in patients with hypophosphatemia and nephrolithiasis	Benign/likely benign

The SLC9A3R1 gene is responsible for encoding the protein Na+/H+ exchange regulatory factor (NHERF1), which plays a crucial role in regulating the function of various membrane transporters, including those involved in phosphate homeostasis. NHERF1 regulates the transport of sodium-phosphate cotransporters, particularly sodium-phosphate cotransporter IIa (NaPi-IIa), to the apical membrane of the proximal convoluted tubules (PCT) in the kidney, resulting in phosphate reabsorption in the PCT. NHERF1 also interacts with other proteins and signalling pathways, including parathyroid hormone and Wnt-β-catenin signalling [6]. A mutation in SLC9A3R1 can impair the function of these cotransporters, resulting in renal phosphate wasting and hypophosphatemia [8]. Hypophosphatemia stimulates the production of 1,25-dihydroxyvitamin D (calcitriol) via upregulation of 1α-hydroxylase in the kidney. Elevated levels of calcitriol increase intestinal calcium absorption, leading to hypercalcemia. With the rise in serum calcium levels, the PTH levels are suppressed through negative feedback. Although PTH levels are suppressed, the elevation in calcitriol levels continues to drive increased calcium absorption in the intestine, leading to PTH-independent hypercalcemia. NHERF1 also interacts with proteins involved in calcium transport in the kidney, such as the transient receptor potential vanilloid 5 (TRPV5) channel [11]. A mutation in SLC9A3R1 could potentially affect calcium reabsorption in the kidneys, further contributing to hypercalcemia. In response to hypercalcemia, the kidneys attempt to excrete the excess calcium via urine, which leads to hypercalciuria. With hypercalciuria, there is an increased amount of free calcium in the urine. When urine becomes supersaturated with calcium, it promotes the nucleation, growth, and aggregation of calcium-containing crystals, eventually forming stones. Low phosphate levels reduce the ability of phosphate to bind to calcium in the urine. This further increases the amount of unbound calcium, making it more likely to crystallize and form stones. Excessive calcium in the renal tubules may deposit in the renal parenchyma, leading to nephrocalcinosis and further predisposing the patient to stone formation.

Inactivating mutation in the SLC9A3R1 gene on chromosome 17 at position 17q25.1 causes NPHLOP2 and has an autosomal dominant mode of inheritance. Till now, 64 NPHLOP2 cases with 17 germline mutations in the SLC9A3R1 gene have been reported [7]. The mutation detected in the current case (c.766C>T; p.Pro256Ser) has not been found in the 1000 Genomes Project, gnomAD v3.1, and gnomAD v2.1 databases. The effect of this variant was evaluated by using in silico analysis by SIFT, LRT, MutationTaster2, and PolyPhen-2 to predict the pathogenic potential, which showed the variant to be possibly damaging. However, due to a lack of sufficient literature evidence and parental segregation studies, this SLC9A3R1 variant was classified as a variant of uncertain significance and requires clinical correlation.

Diagnosing NPHLOP2 requires a high index of suspicion, especially in infants presenting with non-specific symptoms such as failure to thrive or recurrent vomiting. The presence of hypercalcemia, hypophosphatemia, phosphaturia, suppressed PTH, and high 1,25-dihydroxyvitamin D levels in an infant with a genetic mutation in the SLC9A3R1 gene is diagnostic of the condition.

Management of NPHLOP2 should be individualized based on the severity of hypercalcemia and the presence of complications such as nephrolithiasis and bone loss. Regular monitoring and long-term follow-up are essential for optimal outcomes. Acute management of hypercalcemia includes administration of intravenous fluids (0.9% NaCl) and bisphosphonates. Normal saline should be given at a rate of 200-300 mL/m^2^/hour to maintain hydration and correct volume depletion. In severe cases, bisphosphonates like pamidronate (0.5-1 mg/kg I.V infusion over four to six hours) may be used [12]. Others have also used zoledronic acid at a dose of 0.025 mg/kg I.V infusion over one hour [13]. Repeat doses of bisphosphonates may be given if hypercalcemia persists. Although zoledronic acid has not been used widely in infantile hypercalcemia compared to pamidronate, we preferred using zoledronic acid because it is more potent, has a longer duration of action, and requires a shorter infusion time. Our patient developed transient hypocalcemia after receiving zoledronic acid infusion, which is attributable to the abrupt inhibition of osteoclast-mediated bone resorption, resulting in reduced calcium efflux from bone and a temporary imbalance between skeletal calcium uptake and systemic calcium availability. Hemodialysis is reserved for life-threatening hypercalcemia or in cases of renal failure. Some studies have reported treatment options for vitamin D metabolism abnormalities with the use of triazole drugs and rifampicin. Ketoconazole inhibits 1α-hydroxylase activity and reduces 1,25(OH)2D3 production at a dose of 3-10 mg/kg/day. Fluconazole is an alternative with fewer side effects and given at a dose of 3-12 mg/kg/day [14]. Rifampicin may be used to induce CYP3A4, enhancing vitamin D catabolism at a dose of 10-20 mg/kg/day [15]. Patients with persistent hypercalciuria and recurrent nephrolithiasis can be treated with thiazide diuretics.

Phosphate supplementation is often necessary to correct underlying hypophosphatemia. The recommended dose of oral phosphate for infants is generally between 2 and 3 mmol/kg/day, given in multiple daily divided doses to ensure a steady serum phosphate level and minimize gastrointestinal side effects, such as diarrhea or abdominal discomfort [16].

The present study has a few limitations; for example, parental segregation analysis or functional studies have not been performed.

## Conclusions

In infants presenting with PTH-independent hypercalcemia and hypophosphatemia, genetic testing is a valuable tool for identifying the etiology. Mutations in the SLC9A3R1 gene should be part of the genetic panel for patients with infantile hypercalcemia and hypophosphatemia. Long-term phosphate supplementation and cautious use of bisphosphonates in an acute setting constitute the treatment of NPHLOP2.
